# Potential Impacts of *Roe v. Wade* repeal on Sexual and Reproductive Health in Sub‐Saharan Africa

**DOI:** 10.1002/puh2.88

**Published:** 2023-06-01

**Authors:** Olabode Ekerin, Deborah Oluwaseun Shomuyiwa, Alex Vandy Saffa Bayoh, Ouma Sarah Atieno, Yusuf Sheku Tejan, Antor O. Ndep, Dawit Tesfagiorgis Mengesha, Mat Lowe

**Affiliations:** ^1^ Lagos State University College of Medicine Lagos Nigeria; ^2^ The WiseUp Initiative for Good Health and Community Development Kaduna Nigeria; ^3^ Faculty of Pharmacy University of Lagos Lagos Nigeria; ^4^ Global Health Focus Lagos Nigeria; ^5^ ICAP Columbia University Freetown Sierra Leone; ^6^ Maseno University School of Medicine Kisumu Kenya; ^7^ Ministry of Health and Sanitation Freetown Sierra Leone; ^8^ College of Medicine and Allied Health Sciences University of Sierra Leone Freetown Sierra Leone; ^9^ Department of Public Health, Faculty of Allied Medical Sciences, College of Medical Sciences University of Calabar Calabar Nigeria; ^10^ St. Paul's Hospital Millennium Medical College Addis Ababa Ethiopia; ^11^ Society for the Study of Women's Health Kanifing The Gambia

**Keywords:** abortion, maternal mortality, reproductive health, sexual health, sub‐Saharan Africa, Roe v. Wade, USA

## Abstract

The United States (US) Supreme Court's decision to repeal *Roe v. Wade* has had significant implications for abortion care in the US, especially for emergency care. This ruling may also have an impact on sub‐Saharan Africa, where restrictive laws and policies limit women's access to safe and legal abortions. Sub‐Saharan Africa is the region with the highest rate of unwanted pregnancies and unsafe abortions, resulting in 15,000 preventable deaths annually. Although every country in sub‐Saharan Africa has at least one legal basis for abortion, they remain limited. In Nigeria, where 29% of pregnancies are unintended, 48% of these led to abortions, amounting to over 1.4 million abortions between 2015 and 2019. Unsafe abortions are prevalent in the region due to socioreligious stigma, particularly for unmarried women. Criminal charges can result from violating these limitations, with prison terms ranging from 3 to 14 years. The reversal of *Roe v. Wade* could have significant consequences in abortion‐related policies and politics in sub‐Saharan Africa, encouraging nations to adopt restrictive policies and legitimizing them. The landmark decision of *Roe v. Wade* may also impact the rate of unsafe abortions in the region, much like the US Mexico City Policy. With the high probability of foreign assistance funds for safe abortions depleted, sub‐Saharan African countries must prepare for this financial vacuum in health response. Therefore, the repeal of *Roe v. Wade* may lead to increased maternal morbidity and mortality in sub‐Saharan Africa, where access to safe and legal abortions is already limited.

## INTRODUCTION

After almost 50 years, the recent United States (US) Supreme Court decision on *Dobbs v. Jackson Women's Health Organization* repealing *Roe v. Wade* may eliminate the legal right to abortion in some states in the US. Although the ruling was issued in the US, its ripple effect will be felt in parts of the world, especially sub‐Saharan Africa, where some countries have restrictive laws that limit women's access to abortions with exceptions only in circumstances of rape or incest or to save a woman's life [1]. *Dobbs v. Jackson* concerns state legislation in Mississippi that outlawed abortion beyond 15 weeks. Because *Roe v. Wade* protected a pregnant person's right to an abortion before fetal viability, often regarded as approximately 24 weeks, a Mississippi court invalidated the statute as illegal [2]. Mississippi challenged the lower court's decision, and the matter ended before the US Supreme Court. For the first time in almost 50 years, the court addressed the validity of prohibiting abortion before viability in *Dobbs v. Jackson* [2]. This ruling describes a significant shift in reproductive health legislation in the US.

*Roe v. Wade* lawsuit was commonly referred to simply as “Roe,” after the plaintiff, Norma McCorvey, also known by the pseudo name “Jane Roe.” Henry Wade, the defendant at the time, was the District Attorney for Dallas County (Texas) [2]. Roe overturned legislation that rendered abortion unconstitutional in some US states, ruling that abortion is permitted until the fetus is viable or when a baby may survive outside the uterus. At the time of the ruling, fetal viability was typically considered to be around 28 weeks, but medical advancements have since reduced this to 24 weeks [2]. The repeal of *Roe v. Wade* translates that abortion laws will now be “state‐prescribed.”

States in the US have been divided on abortion stands. Although Indiana's legislature has adopted hundreds of abortion‐related bans during the last decade, a 15‐week abortion restriction was enacted in Florida in July 2022 [3]. California, New York, Oregon, and Washington have codified abortion rights into state law. Others, like Iowa, Minnesota, and Montana, have acknowledged the right in court [3]. However, this does place a constitutional barrier to access to abortion as the capacity of a person to obtain an abortion is determined by the state they live. Repealing *Roe v. Wade* may embolden restrictive abortion laws in sub‐Saharan Africa, leaving women without access to safe and legal abortions. This commentary explores the potential impact of the repeal on reproductive health in sub‐Saharan Africa.

## ABORTION IN SUB‐SAHARAN AFRICA

Abortion is one of the most controversial public health issues in sub‐Saharan Africa. Abortion is riskier in sub‐Saharan Africa than in any other region globally, accounting for about 15,000 preventable deaths annually [4]. Before the twenty‐first century in sub‐Saharan Africa, only South Africa had legalized abortion. It was long considered a moral and theological matter until 1994, when it was recognized as a public health crisis that needed to be addressed during the International Conference on Population and Development (ICPD) in Cairo, Egypt [5]. Following the ICPD, there was a significant shift in response to abortion due to policy advocacy and increased awareness of abortion care, which led to Africa's commitment to eradicating abortion‐related morbidity and mortality. [5] Although every country in sub‐Saharan Africa has at least one legal basis for abortion, these remain limited. Nigeria, the most populous African nation, has two of the most stringent abortion laws in Africa. The penal code (Northern Nigeria) and the criminal code (Southern Nigeria) allow abortion only if the mother's life is in danger [6]. Benin has the most liberal abortion laws in Africa [7], but its impact on other sub‐Saharan African countries may not be significant.

In sub‐Saharan Africa, nearly all abortions are a result of unintended pregnancies [4]. The region has the world's highest unwanted pregnancy rate, at 91 per 1000 women, owing to the generally high pregnancy rate (218 per 1000) [4, 8]. In Nigeria, from 2015 to 2019, 29% of pregnancies were unintended, and 48% of these led to abortions. These amount to over 1.4 million abortions within that 5‐year period [9]. Unfortunately, nearly half of all abortions performed in sub‐Saharan Africa are unsafe and contribute to maternal morbidity and mortality [4]. Despite the high risk associated with unsafe abortions, most countries in the region prohibit abortions except in cases where the pregnant woman's life is in danger. Criminal charges can result from violating these limitations, with prison terms ranging from 3 to 14 years [7, 8]. Legal channels to access abortions are also bypassed due to socioreligious stigma, particularly for unmarried women [4]. However, areas that allow abortions under broader circumstances have fewer unsafe abortions and associated fatalities [5].

## POSSIBLE IMPACT OF THE REPEAL

The *Roe v. Wade* reversal could have significant consequences in the abortion‐related policies and politics in the sub‐Saharan region [10]. Weakening abortion rights globally, the shift may encourage nations to follow suit and adopt restrictive policies, pointing to the US as a legitimizing force. Such effects are reminiscent of the ripple effect caused by the US Mexico City Policy in sub‐Saharan Africa [10, 11]. Similarly, the landmark decision of *Roe v. Wade* may impact the rate of unsafe abortions in the region, much like the Mexico City Policy.

### Impact on abortion rate

According to a study, the US Mexico City Policy, often known as the global gag rule (GGR) by critics, has been connected to increased abortion rates in sub‐Saharan African countries [11]. Abortions increased significantly in sub‐Saharan Africa among women affected by the Policy. This increase was discovered to be accompanied by a decrease in the use of modern contraception and an increase in pregnancies. After this restriction was abolished, the pattern of increased abortions and decreasing contraceptive usage reversed.

The alternating patterns during periods when the Policy is in place during the administrations of the Republican Party and when the Policy is rescinded during the administrations of the Democratic Party strengthen the evidence for the Policy's function and suggest that the Policy's effects are reversible [10, 11]. Another research discovered that throughout the period the Policy was in effect, abortion rates did not drop for any demographic. In fact, the chance of abortion for women in Ghana's rural areas increased by 50%–60% [12]. Additional unplanned births disproportionately impacted the most vulnerable women, who could not access abortion services.

### Impact on sexual and reproductive health services

Similar to the GGR, *Roe v. Wade* has the potential to impact other services that will influence the decrease in unsafe abortion. A study conducted in Nepal, Kenya, and Zambia revealed that the US Mexico City Policy negatively impacted family planning, maternal and child health, and human immunodeficiency virus services, leading to more unplanned pregnancies [13]. Given the impact that GGR has had in these countries, the effect of *Roe v. Wade* may be severe in similar regions.

Unmet demand for modern contraception and the percentage of unplanned pregnancies in Uganda are quite high compared to other low‐ and middle‐income countries (unmet need: 20% vs. 13%; unintentional births: 41% vs. 23%), and approximately 14% of all pregnancies result in abortion [14]. In this country, safe abortion management is prohibited, and conflicting abortion law creates a complex legal environment for abortion providers. In Nigeria, abstinence‐only sex education is preferred by cultural and religious stakeholders, whereas young girls and women are often victims of rape, early marriages, and high birth rates. These, combined with a low contraceptive uptake rate of 15% or less, lead to high rates of unwanted pregnancies [15]. Due to tight legislation in several sub‐Saharan African countries, most women and girls seek abortions through unskilled and clandestine practitioners, which results in negative consequences [1].

### Impact on achieving the SDGs

The SDGs set a target for governments to “reduce the global maternal mortality ratio to fewer than 70 per 100,000 live births by 2030” (Target 3.1) [16]. However, unsafe abortions remain a significant contributor to maternal mortality, particularly in sub‐Saharan Africa, posing a challenge to achieving this target. In addition, the restrictive laws on abortion, such as those introduced in the US with the *Roe v. Wade* repeal, can potentially limit comprehensive reproductive healthcare services. This limitation could escalate the number of unsafe abortions, ultimately jeopardizing the achievement of the SDGs.

### Impact on US foreign assistance funds

The repeal of *Roe v. Wade* may impact US financing and Policy on sexual and reproductive health and rights in (SRHR) in sub‐Saharan Africa. The US administration represents big funders of the SRHR programs in sub‐Saharan Africa [17]. Advocates worry that the repeal would lead to cuts in the US supports for family planning, sexual education, and gender‐related public health initiatives [18]. A study found that countries in sub‐Saharan Africa receiving more financial aid were disproportionately affected by the GGR, leading to a higher rate of induced abortions among women in these nations [8]. *Roe v. Wade* does not directly influence US foreign policy, but its repeals would signal the US’ stance on reproductive healthcare.

### Impact on advocacy

There is also fear that repealing *Roe v. Wade* would strengthen the anti‐choice campaign in sub‐Saharan Africa. It might further stir up antiabortion and anti‐right women's sentiments and set a precedent to reconsider the measures governments will take on SRHR issues, which may increase the stigma associated with abortion across countries in sub‐Saharan Africa.

## CONCLUSION

The repeal of *Roe v. Wade* can potentially impede progress toward reproductive rights and healthcare access in sub‐Saharan Africa. It could strengthen the stance of conservative countries and hinder the progress toward liberalizing abortion laws. Despite concerns over the potential negative impact of this ruling, global health and reproductive rights advocates must prioritize evidence‐based approaches to policy and funding choices. Additionally, governments and stakeholders of SRHR in sub‐Saharan Africa must take urgent action to step up and fill the financial void that may result from the repeal of this landmark decision. Ultimately, it is essential to prioritize the health and well‐being of individuals and communities globally and collaborate to ensure access to safe and comprehensive reproductive healthcare.

## AUTHOR CONTRIBUTIONS

Olabode Ekerin wrote conducted the literature search and prepared the first draft. Olabode Ekerin, Deborah Oluwaseun Shomuyiwa, Alex Vandy Saffa Bayoh, Ouma Sarah Atieno, Yusuf Sheku Tejan, Dawit Tesfagiorgis Mengesha, Antor O. Ndep, and Mat Lowe edited, reviewed the draft, and prepared the final. All authors reviewed the final draft for publication.

## CONFLICT OF INTEREST STATEMENT

Deborah Oluwaseun Shomuyiwa is a Youth Editorial Board member, and Mat Lowe is an Editorial Board member of Public Health Challenges and coauthors of this article. To minimize bias, they have been excluded from all editorial decision‐making processes related to the acceptance of this article for publication.

## References

[1] Okonofua FE . Roe vs. Wade: US prevarication poses threat to sub‐Saharan Africa. Afr J Reprod Health. 2022;25:5‐13. https://ajrh.info/index.php/ajrh/article/view/3316 10.29063/ajrh2022/v26i5.1 37585092

[2] Roe vs. Wade 410 US. 113. Justia Opinion Summary and Annotations. 1973. Available from https://supreme.justia.com/cases/federal/us/410/113/

[3] Guttmacher Institute December 2022: State Policy Trends 2022: In a Devastating Year, US Supreme Court's Decision to Overturn Roe Leads to Bans, Confusion and Chaos. Available from https://www.guttmacher.org/2022/12/state‐policy‐trends‐2022‐devastating‐year‐us‐supreme‐courts‐decision‐overturn‐roe‐leads

[4] Gebremedhin M , Semahegn A , Usmael T , Tesfaye G . Unsafe abortion and associated factors among reproductive aged women in sub‐Saharan Africa: a protocol for a systematic review and meta‐analysis. Syst Rev. 2018;7(1):130. doi: 10.1186/s13643-018-0775-9 30144826 PMC6109307

[5] Benin Passed One of Africa's Most Liberal Abortion Laws. Why are Women Still Dying? The Guardian. Available from https://www.theguardian.com/global‐development/2023/feb/28/benin‐africa‐liberal‐abortion‐laws‐women‐still‐dying

[6] Onyemelukwe C . Abortion law in Nigeria. Bey Health Q. 2018; Issue 2, 17:16, pp. 34‐38.

[7] Guttmacher Institute December 2020: Report From Unsafe to Safe Abortion in Sub‐Saharan Africa: Slow but Steady Progress Akinrinola Bankole, Lisa Remez, Onikepe Owolabi, Jesse Philbin, and Patrice Williams. Available from https://www.guttmacher.org/report/from‐unsafe‐to‐safe‐abortion‐in‐subsaharan‐africa

[8] Bendavid E , Avila P , Miller G . United States aid policy and induced abortion in sub‐Saharan Africa. Bull World Health Organ. 2011;89(12):873‐880C. doi: 10.2471/BLT.11.091660 22271944 PMC3260902

[9] Guttmacher Institute, Undefined Country Profile. 2022, https://www.guttmacher.org/regions/africa/nigeria

[10] Jones KM . Contraceptive supply and fertility outcomes: evidence from Ghana. Econ Dev Cult Change. 2015;64:31‐69. doi: 10.1086/682981

[11] Macklin R . The “global gag rule”: curtailing women's reproductive rights. Indian J Med Ethics. 2019;4(3):198‐201. doi: 10.20529/IJME.2019.015 31213424

[12] Giorgio M , Makumbi F , Kibira SPS , Bell S , Anjur‐Dietrich S , Sully E . Investigating the early impact of the Trump Administration's Global Gag Rule on sexual and reproductive health service delivery in Uganda. PLoS One. 2020;15(4):e0231960. doi: 10.1371/journal.pone.0231960 32343713 PMC7188216

[13] Generation Equality? Trends from a Decade of Donor Funding for SRHR. https://donortracker.org/insights/generation‐equality‐trends‐decade‐donor‐funding‐srhr

[14] Kalila J , Johnson Z . Generation Equality? Trends from a Decade of Donor Funding for SRHR. 2021. Available from https://donortracker.org/insights/generation‐equality‐trends‐decade‐donor‐funding‐srhr

[15] WHO . The Global Health Observatory. Accessed 10th March 2022. https://www.who.int/data/gho/data/themes/topics/indicator‐groups/indicator‐group‐details/GHO/maternal‐mortality

[16] Bankole A , Remez L , Owolabi O , Philbin J , Williams P . From Unsafe to Safe Abortion in Sub‐Saharan Africa: Slow but Steady Progress. Guttmacher Institute; 2020:1‐45. https://www.guttmacher.org/report/from‐unsafe‐to‐safe‐abortion‐in‐subsaharan‐africa

[17] Brooks N , Bendavid E , Miller G . USA aid policy and induced abortion in sub‐Saharan Africa: an analysis of the Mexico City Policy. Lancet Glob Health. 2019;7(8):e1046‐e1053. 10.1016/S2214-109X(19)30267-0 31257094

[18] Musa A . Sex education in Nigeria: attitude of secondary school adolescents and the role of parents and stakeholders. Open J Educ Dev. 2020;1(1):1‐30. doi: 10.52417/ojed.v1i1.60 (ISSN: 2734‐2050)

